# Transorbital neuroendoscopic approach for recurrent sinonasal inverted papilloma

**DOI:** 10.3171/2025.1.FOCVID24201

**Published:** 2025-04-01

**Authors:** Jason R. Crossley, Hussam Abou-Al-Shaar, S. Tonya Stefko, Paul A. Gardner, Garret Choby

**Affiliations:** Departments of 1 Otolaryngology–Head and Neck Surgery,; 2 Neurosurgery, University of Pittsburgh Medical Center, Pittsburgh, Pennsylvania and; 3 Ophthalmology, University of Pittsburgh Medical Center, Pittsburgh, Pennsylvania

**Keywords:** transorbital surgery, inverted papilloma, skull base surgery

## Abstract

This surgical video demonstrates the case of a 38-year-old male with recurrent inverted papilloma involving the lamina papyracea and frontal sinus outflow tract. The frontal sinus was diminutive on the side of the tumor, and as such a combined endoscopic endonasal with endoscopic transorbital via transcaruncular route was advised for resection of the anterolateral tumor and contralateral margin. After endonasal resection, a transorbital approach was utilized to traverse the lamina papyracea from lateral to medial and resect tumor along the ethmoid roof and posterior table of the frontal sinus.

The video can be found here: https://stream.cadmore.media/r10.3171/2025.1.FOCVID24201

## Transcript

### 0:20 Case Overview.

In this surgical video, we will be demonstrating a transorbital neuroendoscopic approach for recurrent sinonasal inverted papilloma. The patient is a 38-year-old male with a recurrent inverted papilloma following sinus surgery in the community setting. Several months later, recurrence was noted along the orbit extending to the frontal sinus outflow tract. In this preoperative CT, recurrent tumor can be seen along the lamina papyracea, frontal sinus outflow tract, and ethmoid roof on the right. Here, a T1 postcontrast MRI in coronal view again demonstrates the tumor and also shows involvement of an interfrontal sinus cell. A combined transorbital and endonasal approach was selected over an endonasal approach alone in this case due to the frontal sinus anatomy and tumor location in this patient. Specifically, a narrow anterior-posterior diameter of the right frontal sinus outflow tract measured here in a preoperative CT in the sagittal plane at 7.4 mm limits access to the inferior aspect of the anterior table, even if extended drilling of the nasofrontal beak were to be carried out. Demonstrated in red here is the area of the nasofrontal beak and anterior table, which are possible tumor attachment sites which would not be accessible from an endonasal route alone.

Here, a preoperative CT in the axial plane is shown. This patient has an interfrontal sinus cell which may be involved by tumor. The anterior and left lateral aspects of this cell are bordered in red. Approaching this cell transorbitally, as demonstrated with the blue arrow, provides a more direct lateral to medial view than an endonasal approach alone. This trajectory is advantageous for viewing and addressing tumor attachments here. In a coronal view, the limited lateral extent of the right frontal sinus is demonstrated here by a blue arrow. This limited lateral extent is better approached by a transcaruncular over an eyelid incision because the caruncle is situated more medial and thus more directly over the tumor than the eyelid, which may extend unnecessarily lateral for this patient’s frontal sinus anatomy. An additional advantage of the transcaruncular route is avoidance of a visible scar. A potential downside of this approach is persistent edema and irritation at the incision site.^1^

### 2:39 Beginning of Case With Endonasal Approach.

We begin the case with a biopsy of the recurrent tumor along the lamina to confirm the diagnosis. This also helps rule out squamous cell carcinoma, which would be managed more aggressively and is a tumor that inverted papilloma may transform into.^2^ Frozen was consistent with inverted papilloma without evidence of dysplasia. Here, the right maxillary sinus, papilloma along the lamina papyracea, and septum are labeled. First, cold steel is used to debulk tumor along the lamina, and then here we use a microdebrider to remove tumor from the lamina as well as the frontal sinus outflow tract here, as well as inflammatory mucosa. Monopolar cautery is used to develop a plane through the mucosa down to the bone of the lamina papyracea. This is extended along the orbital floor inferior to the visible tumor, and then a curette is used to elevate this mucosa with involved tumor from the underlying bone. Here we use the microdebrider again to debulk the tumor and inflammatory polyps in the frontal sinus outflow tract, which is seen here. A Hosemann punch is used to enlarge the frontal sinus outflow tract anteriorly. We know this has a variant of normal anatomy in the frontal sinus which is small and only extends minimally over the orbital roof. Again, here are more adherent tumor is removed from the lamina papyracea, which is viewed. At this point, we then begin drilling along the lamina papyracea to remove any tumor which may be involved in the underlying bone there. An Aquamantys bipolar is used here to coagulate mucosa along the periphery of where the tumor had been resected.

### 3:15 Demonstration of Transorbital Approach.

Here a transcaruncular approach is demonstrated. Following injection of local anesthesia to the caruncle, an incision is made between the plica and the caruncle. Using a combination of sharp and blunt dissection, this incision is carried down medially to the level of the posterior lacrimal crest. The periorbita is then incised at the level of the posterior lacrimal crest. Once this is done, periosteum is elevated posteriorly to prevent prolapse of orbital fat into the surgical wound. A corneal shield and then tarsorrhaphy sutures are placed. A SPIWAY device is inserted here. This allows retraction of orbital contents and passage of three instruments including an endoscope transorbitally.^3,4^ At this point we have done our transorbital transcaruncular approach and a Kerrison rongeur is being used to remove bone from the orbital roof. Now dura of the anterior fossa is seen. Then, from this view you can see anteriorly to this, the lamina in continuity with the sinonasal cavity as seen on the right side of the screen here. Pledgets which have been placed in the nasal cavity are also seen. Here we can see drilling along the anteromedial aspect of the orbital roof as well as the bone along the frontonasal duct. Possibly involved mucosa is also elevated here sharply. Here, Kerrison rongeurs are being used to remove the lamina papyracea in a posterior to anterior fashion. Here we’re removing mucosa from the contralateral frontal sinus. The transorbital approach permits access to the frontal sinus pneumatization over the orbit as well as the medial frontal sinus toward the intersinus septation. This access would not be possible from a solely endonasal approach. At this point, a small CSF leak is noted from the first olfactory fiber.

### 7:27 Repair of CSF Leak and Packing Placement.

The Aquamantys bipolar is used here to coagulate mucosa around the periphery of the tumor after the resection. We’re also bipolaring mucosa along the CSF leak site to help with the adherence of our free mucosal graft, which is now being placed over the ethmoid roof CSF leak site. Here an ethmoid is being used to position the free mucosal graft transnasally, and now a Frazier tip suction is being placed transorbitally to help lay the free graft appropriately. This free mucosal graft was harvested from the right nasal floor. After the graft is in good location over the leak, Gelfoam is placed followed by Vectra F. This is absorbable packing which can help maintain the patency of the frontal sinus outflow tract.^5^ NasoPore is then used to support the free mucosal graft. The transcaruncular incision was closed with two inverted 6-0 Vicryl sutures. This concludes the procedure. The patient is observed in the hospital overnight.

### 8:36 Review of Postoperative CT.

Postoperative CT scan demonstrates no evidence of residual tumor along the orbit or frontal outflow tract.

## Disclosures

The authors report no conflict of interest concerning the materials or methods used in this study or the findings specified in this publication.

## Author Contributions

Primary surgeon: Gardner, Choby. Assistant surgeon: Crossley, Stefko. Editing and drafting the video and abstract: Crossley, Stefko, Gardner, Choby. Critically revising the work: Crossley, Abou-Al-Shaar, Gardner, Choby. Reviewed submitted version of the work: Crossley, Abou-Al-Shaar, Gardner. Approved the final version of the work on behalf of all authors: Crossley. Supervision: Crossley, Choby.

## Supplemental Information

### Patient Informed Consent

The necessary patient informed consent was obtained in this study.
